# Optimal Biomechanical Performance in Upper-Limb Gestures Depends on Velocity and Carried Load

**DOI:** 10.3390/biology11030391

**Published:** 2022-03-02

**Authors:** Cristina Brambilla, Matteo Malosio, Gianluigi Reni, Alessandro Scano

**Affiliations:** 1 Institute of Intelligent Industrial Systems and Technologies for Advanced Manufacturing (STIIMA), Italian Council of National Research (CNR), Via Previati 1/E, 23900 Lecco, Italy; cristina.brambilla@stiima.cnr.it (C.B.); matteo.malosio@stiima.cnr.it (M.M.); 2 Bioengineering Laboratory, Scientific Institute, IRCCS “Eugenio Medea”, 23842 Bosisio Parini (Lecco), Italy; gianluigi.reni@lanostrafamiglia.it

**Keywords:** power, energy, torque, upper limb, industry, fatigue

## Abstract

**Simple Summary:**

In the last few years, the physical health and mental health of workers in an industrial context while interacting with collaborative robots have become of primary interest in the research field. The characterization of the mental and psychophysical health of workers and subjects can be investigated with biomechanical analysis. In this work, a biomechanical model was used to perform simulations of the biomechanics of reaching against gravity movements as a paradigmatic motion primitive underlying several functional tasks. Motion tracking data were acquired with motion capture, and, in a simulation environment, multiple movement speeds and carried loads were changed in order to analyze the effects on upper-limb kinematics and dynamics. An optimal range of velocities for human motion was found in which the expended energy was lower. The deviation from energy optimum for the upper limb was evaluated when testing nonoptimal movement conditions. These results can be useful in a human–robot collaboration scenario to tune setup parameters to preserve the physical and mental health of the workers, to assess biomechanics, and to define fatigue and effort indices.

**Abstract:**

In the last few years, there has been increased interest in the preservation of physical and mental health of workers that cooperate with robots in industrial contexts, such as in the framework of the European H2020 Mindbot Project. Since biomechanical analysis contributes to the characterization of the subject interacting with a robotic setup and platform, we tested different speed and loading conditions in a simulated environment to determine upper-limb optimal performance. The simulations were performed starting from laboratory data of people executing upper-limb frontal reaching movements, by scaling the motion law and imposing various carried loads at the hand. The simulated velocity ranged from 20% to 200% of the original natural speed, with step increments of 10%, while the hand loads were 0, 0.5, 1, and 2 kg, simulating carried objects. A 3D inverse kinematic and dynamic model was used to compute upper-limb kinematics and dynamics, including shoulder flexion, shoulder abduction, and elbow flexion. An optimal range of velocities was found in which the expended energy was lower. Interestingly, the optimal speed corresponding to lower exerted torque and energy decreased when the load applied increased. Lastly, we introduced a preliminary movement inefficiency index to evaluate the deviation of the power and expended energy for the shoulder flexion degree of freedom when not coinciding with the minimum energy condition. These results can be useful in human–robot collaboration to design minimum-fatigue collaborative tasks, tune setup parameters and robot behavior, and support physical and mental health for workers.

## 1. Introduction

Industry 4.0 aims at developing highly automated and digitalized processes with the use of electronics and information technologies in manufacturing and services [1]. Indeed, in the last decade, the constantly growth of Industry 4.0 led to a high level of automatization, increasing the productivity and efficiency in industrial applications [2]. In this scenario, human operators face increased complexity of their daily tasks, and they need to be highly flexible in a dynamic working environment [3]. Therefore, the physical health and mental health of workers are becoming of great interest in the research field. To sustain physical strain during highly repetitive tasks, gravity-assistive wearable upper-limb exoskeletons have been introduced to improve conditions for workers and to reduce risks for workers’ ergonomics [4]. Furthermore, new workspaces have been developed in which human workers cooperate with robots [5]. In this scenario, the physical safety of the worker is of primary importance [6] and the quality of experience and level of engagement of operators working with collaborative robots (cobots) have also become a central issue in the research field. In order to improve working experience, it is necessary that the task is challenging enough to require concentration, engagement, and exploitation of personal skills and resources [7]; otherwise, the subject could feel boredom and apathy [8]. On the other hand, excessive physical demand could affect the mental state of the worker, reducing the cognitive condition and inducing unsafe behaviors and higher risks [9]. Physical health and mental health are strictly related to one another; physical fatigue affects mental state, while mental fatigue impairs physical performance [10]. Guaranteeing favorable physical and psychosocial working conditions is fundamental for maintaining mental and physical health [11].

In this regard, the MindBot project (https://www.mindbot.eu/ accessed on 20 February 2022), funded by Horizon2020, was launched to define the guidelines and design a “mental-health-friendly” cobot-based manufacturing workplace. The setup is defined so that the subject is constantly monitored by dedicated tools in four different dimensions: biomechanical, physiological, social, and psychological. The architecture of the experimental platform developed in the MindBot project was described extensively in a previous study [12].

A biomechanical characterization of the operator is needed to assess the physical and mental health of workers so that the robot can adapt to improve human–robot interaction and to identify the presence of fatigue and effort. To do this, we used a 3D inverse kinematic and dynamic model based on a previously implemented biomechanical model [13], which allows the computation of kinematic and dynamic parameters. This model is integrated with data registered using Microsoft Azure Kinect cameras, which estimate human joint positions [14]. The presence of cooperation with a robotic architecture and, in general, with an assembly or working scenario may alter the normal execution of human movement, due to the speed of movements, displaced loads, and other factors. Usually, humans choose the motion trajectory needed to execute a movement that maximizes efficiency, defined according to several optimization criteria [15]. In the literature, how the central nervous system (CNS) selects the best way to move has been highly debated, and different models have been proposed to describe movement efficiency. The minimum jerk model, presented by Flash and Hogan [16], states that the CNS chooses the trajectory that minimizes the integral of the squared jerk (the third derivative of the position), leading to typical bell-shaped velocity profiles. Another model is the minimum torque-change model [17], in which the square of the rate of change of torque integrated over the movement is minimized. The minimum variance model [18], instead, considers that neural commands have signal-dependent noise, hereby the CNS chooses the hand trajectory that minimizes the variance of the position. In 1997, Alexander hypothesized that movement trajectories are chosen to minimize the metabolic energy cost [19]. The metabolic energy cost for arm motion was analyzed using an analytical and a muscle–skeletal model by Zhou et al. [20]; they found trajectories that were similar to those computed by Alexander’s model. Deviations from the optimal criteria induce altered perception of movements and altered biomechanics.

In recent years, Gaveau et al. [21] found that the CNS adapts motor control on both gravitational and inertial constraints. Unlike horizontal arm movements, in which motor control is based mostly on kinematics, in vertical movements the CNS uses a dynamic planning process that integrates a neural representation of the gravity force and exploits gravitational accelerations to reduce muscular activations. In particular, the direction of movement and the orientation of the body axis generate asymmetries in the arm trajectory to compensate for the gravity [22]. Furthermore, Berret et al. [23] proposed that the CNS chooses the movements that minimize the absolute work of forces, including both inertial forces (velocity-dependent) and gravitational forces (direction-dependent). 

The aim of this study was to define the characteristics for the exploitation of the mechanical properties of the upper limb through a biomechanical model and to establish a dataset for the identification of fatigue and inefficiency indices. First, we acquired tracking data from frontal reaching against gravity movements. Subsequently, we performed simulations to assess the modifications of upper-limb biomechanics when spanning through different simulated velocities and simulated carried loads, preserving the natural biomimetic trajectory of the movement. Our aim was to identify a range of speeds in which the expended power and energy are lower and to define a preliminary index for quantifying motor inefficiency on the basis of limb biomechanics. 

## 2. Materials and Methods

**Figure 1:**
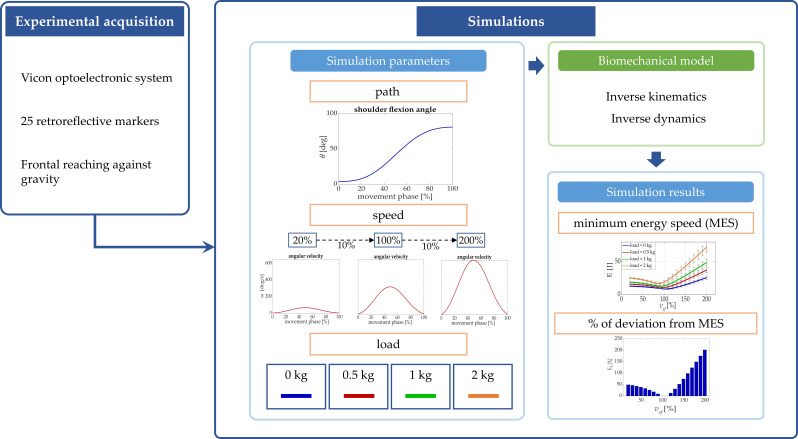
Workflow of the study. Experimental acquisitions were recorded in natural frontal reaching movements. Starting from these data, motion laws were simulated while changing speed of movement and carried load. Simulations were performed with the inverse kinematic and dynamic upper-limb biomechanical model in order to find the range of optimal speed and the percentage of energy and power deviation from the optimum for each simulated condition.

A scheme of the workflow of the study is presented in Figure 1.

### 2.1. Data Acquisition

In order to run the simulations mimicking a physiological profile, two subjects, a male (27 years old; 175 cm; 80 kg) and a female (26 years old; 165 cm; 48 kg), were included in the study with no musculoskeletal impairment affecting performance. Ethical approval was granted by the referring ethical committee (Approval Prot. N. 19/20—CE, 20 April 2020), and the experimental trial was conducted in compliance with the Declaration of Helsinki [24]. The participants provided written informed consent to participate in this study and for the publication of any data included in this article.

Kinematic data were acquired using the Vero Vicon system, a marker-based tracking system consisting of 10 infrared cameras and a set of 25 retroreflective markers placed at anatomical landmarks, following the Vicon Upper-Limb model protocol. Data were pre-elaborated in Nexus software to track and label the markers. The output signals were the trajectories of the markers sampled at 100 Hz and filtered with a third-order Butterworth filter with a cutoff frequency of 6 Hz. Afterward, the data were given as input to the biomechanical model.

The acquired movement was a frontal reaching against gravity. It was chosen since it is a representative movement of the upper limb and can be considered as a motion primitive that allows reaching objects in various contexts, frequently investigated in similar studies. The subject was in standing position and was asked to elevate the right hand at the same height of the shoulder, with the elbow totally extended, aiming at a fixed target. The movement was repeated 10 times at natural speed, which was the same for both subjects. The movement was segmented in forward and backward phases and only the forward phases were considered for the analysis.

### 2.2. Biomechanical Model

**Figure 2:**
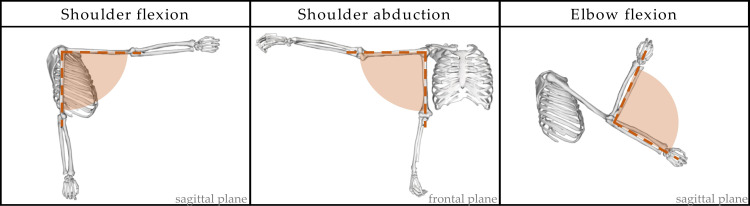
Upper-limb degrees of freedom considered in the analysis: shoulder flexion, shoulder abduction, and elbow flexion.

A 3D inverse dynamics model based on a previously available biomechanical model [13] was used. The model takes as input the 3D trajectory of the markers and the anthropometric parameters, computed with anthropometric tables [25], including the height and weight of the subject. The model allows the computation of kinematic and dynamic parameters of the shoulder, elbow, and wrist joints of both upper limbs, and was also employed for the reconstruction of body volumes, and elaborations on real and simulated data. Three upper-limb degrees of freedom were considered in this study (Figure 2): shoulder flexion and abduction, i.e., the shoulder angle in the sagittal and frontal planes, respectively, and elbow flexion.

For each degree of freedom, the angular profile, the angular velocity, and the angular acceleration were computed. Furthermore, dynamic quantities (torque, power, and energy) were computed using inverse dynamics equations. The power $P_{i}$ exerted at joint level for the degree of freedom i was defined as

(1) $$P_{i}\left(t\right)=\tau_{i}\left(t\right)\omega_{i}\left(t\right),$$

where $\tau_{i}$ is the torque, and $\omega_{i}$ was the angular velocity in each movement repetition *i*. We considered the peak power $P_{i,max}$, computed as the maximum value of the power $P_{i}$.

The expended energy $E_{i}$ was computed as the integral of the power in time.

(2) $$E_{i}=\int_{t_{0}}^{t_{end}}P_{i}\left(t\right)dt,$$

where $t_{0}$ and $t_{end}$ were the initial and the ending time of each reaching movement.

### 2.3. Simulations

Simulations were performed with the recorded data, starting from time series obtained in the experimental acquisition and scaling the velocity of the movement and the carried load.

For the simulations, we used the trajectories recorded in the experimental acquisition to maintain a profile similar to the physiological minimum jerk-like profiles [16] with a bell-shaped velocity profile. Since previous studies showed that the applied inertial and gravitational load may impact the kinematics of the movement [21,22,26], we experimentally compared the free movement and the movement with the proposed loads by computing the correlation coefficients of the velocity profiles and acceleration profiles to verify if, in our setup, we could assume that the kinematics and the velocity profile were the same when changing loads.

Therefore, movement velocity was decreased until 20% of the initial velocity with a step of 10% and increased until 200% with the same step. The velocity scale factor ($v_{sf}$) indicates the scaling of the velocities with respect to the original natural movement.

Since, in industrial contexts, the operator carries loads during assembly processes, different loads were tested. Loads of 0.5 kg, 1 kg, and 2 kg were added to the hand to replicate working conditions found in this scenario in which the worker has to move or displace objects. Combining all the conditions (movement repetitions; scaled velocities; scaled loads), a total of 760 frontal reaching movements were simulated for each subject.

### 2.4. Outcome Measures and Statistics

In order to compare the performance in the different simulated conditions, for each repetition, we considered the maximum torque $\tau_{i,max}$, computed as the maximum of the torque $\tau_{i}$, the peak power $P_{i,max}$, and the expended energy $E_{i}$ for each degree of freedom *i* (shoulder flexion, shoulder abduction, and elbow flexion). The repetitions were taken into consideration separately in order to consider physiological variability. The energetic optimum was considered as the velocity at which the expended energy was minimum in each loading condition. A statistical analysis was performed on the expended energy computed for the shoulder flexion (the leading joint to perform the movement) in order to evaluate which velocities led to significantly different biomechanical outputs. The data distributions were tested for normality through the Kolmogorov–Smirnov test, and the ANOVA test was performed, setting the alpha level of significance at 0.05. Then, the Tukey–Kramer post hoc test was used to identify the significant differences. Lastly, we evaluated how much the peak power and the energy of the shoulder flexion deviated from the optimal point. Peak power deviation $P_{j,\%}$ at velocity j was computed as

(3) $$P_{j,\%}=\frac{P_{j}-P_{opt}}{P_{opt}}\cdot 100,$$

where $P_{j}$ is the peak power at velocity j, and $P_{opt}$ is the peak power at the optimal biomechanical condition (minimum energy speed). Energy deviation $E_{j,\%}$ at velocity j was calculated as

(4) $$E_{j,\%}=\frac{E_{j}-E_{opt}}{E_{opt}}\cdot 100,$$

where $E_{j}$ is the expended energy at velocity j, and $E_{opt}$ is the energy requested at the optimal biomechanical condition (minimum energy speed). *E*_%_ is suggested as an index that quantifies the inefficiency of a movement given a loading condition. 

## 3. Results

**Figure 3:**
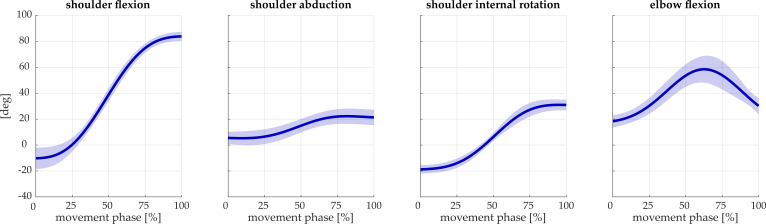
Kinematics of the reaching movements. Articular angles (shoulder flexion, shoulder abduction, shoulder internal rotation, and elbow flexion) are reported as the average for the two subjects. Solid lines represent the mean across repetitions, while the shaded areas show the standard deviations.

To clarify the gesture performed and used in the simulation, the articular angles (shoulder flexion, shoulder abduction, shoulder internal rotation, and elbow flexion) are reported in Figure 3, for both subjects. 

**Figure 4:**
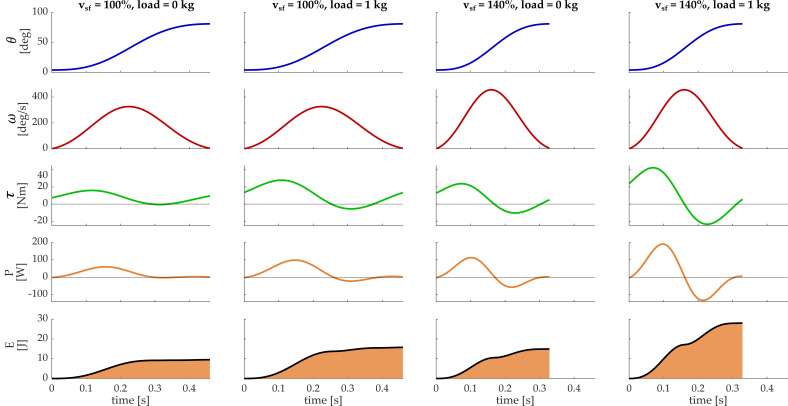
Biomechanics of the reaching movements in different simulated conditions. In the rows, time series of the shoulder flexion angle, angular velocity, torque, power, and energy of one movement repetition are reported. In the columns, four simulated conditions are reported: the first two columns correspond to simulations with a velocity scale factor (*v_sf_*) = 100% and with load = 0 kg and load = 1 kg, respectively; in the last two columns, simulations with a velocity scale factor (*v_sf_*) = 140% and with load = 0 kg and load = 1 kg are reported.

The correlation coefficients of the velocity profiles (when comparing free movement and with loads) were always above 0.975 (mean = 0.982 ± 0.02), and the correlation coefficients of the acceleration profiles were always above 0.963 (mean = 0.971 ± 0.01). Since the movement was standardized and highly repeatable, we could assume that changes in the kinematics (velocity profile) were negligible when changing loads for this specific simulation, even though this assumption cannot be generalized to a generic condition as previously reported [21,26]. In Figure 4, the time series of the outcome measures from the biomechanical model (articular angle, angular velocity, torque, power, and expended energy) are reported for the shoulder flexion with two different velocities—the optimal one (*v_sf_* = 100%) and with *v_sf_* = 140%, and with two loading conditions—load = 0 kg and load = 1 kg, selected from the dataset as two demostrative conditions. 

Referring to Figure 4, since the followed path was the same for each simulation, shoulder flexion angle reached about 81° in all the conditions but the time needed to execute the movement changed depending on the considered velocity. It can be seen that angular velocity had the same bell-shaped profile, but it was stretched in time, whereby a higher peak denoted a lower execution time. Velocity and carried load influenced the dynamic outcomes; upon changing the velocity and/or the loading condition, torque and power had some negative components due to the higher inertia requiring negative torque to decelerate the flexion of the shoulder. 

**Figure 5:**
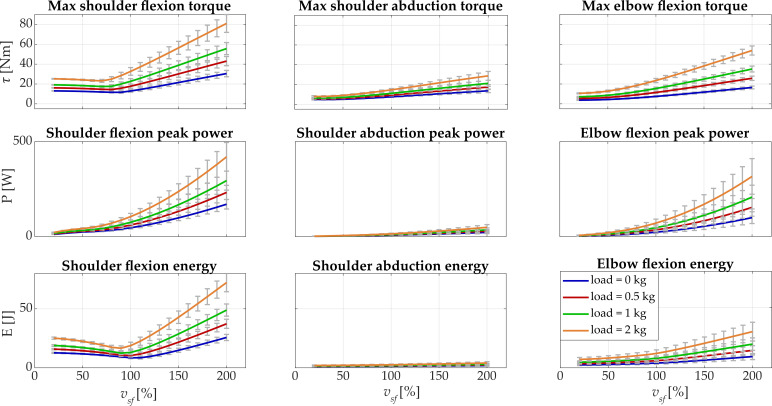
Results of the simulations when changing velocity and carried load are reported for the male subject. For each *v_sf_* and load, the means across repetitions are shown, while the standard deviations are represented with gray error bars. In the first row, the maximum torque is depicted for all the considered upper-limb degrees of freedom; in the second row, the peak power is reported; the expended energy is presented in the last row. In each column, the considered degrees of freedom are represented (shoulder flexion, shoulder abduction, and elbow flexion).

In Figure 5, the recruiting curves of the maximum torque, the peak power, and the expended energy when changing the velocity of the movement and the loading condition are reported for the male subject. 

Shoulder flexion was the degree of freedom that showed the greatest range of motion during the execution of the movement and exhibited significant differences among the tested conditions. The maximum torque had a range of 13.1–25.2 N·m at *v_sf_* = 20% and changed minimally until *v_sf_* = 80%. When increasing velocity, the maximum torque increased about linearly reaching the maximum value at the highest velocity: 30.5 N·m (0 kg), 43.0 N·m (0.5 kg), 55.6 N·m (1 kg), and 80.9 N·m (2 kg). The peak power started in the range 11.5–21.9 W at *v_sf_* = 20% and reached the maximum values at *v_sf_* = 200%: 170.2 W (0 kg), 232.3 W (0.5 kg), 294.6 W (1 kg), and 420 W (2 kg). At *v_sf_* = 20%, the expended energy was between 12.7 J (0 kg) and 25.1 J (2 kg) and the maximum values were found at the highest velocity: 25.7 J (0 kg), 37.3 J (0.5 kg), 48.9 J (1 kg), and 72.3 J (2 kg). In all the loading conditions, the expended energy of the shoulder flexion had a minimum point that was identified as the optimal velocity in which the motor efficiency (lower energy needed) was higher. For load = 0 kg and load = 0.5 kg, the optimal velocity was 158.2 °/s (*v_sf_* = 100%), which corresponded to an expended energy of 8.5 J and 10.4 J; for load = 1 kg and load = 2 kg, the optimum was found at 142.5 °/s (*v_sf_* = 90%) with E = 12.5 J and E = 16.6 J. 

Since the shoulder abduction was the degree of freedom that was least involved in the movement (small range of motion, expected since the movement is frontal), the changes among conditions were very low. Maximum torque was lower than 8 N·m for *v_sf_* = 20% in all the conditions and reached 13.6 N·m (0 kg) and 28.7 N·m (2 kg) at *v_sf_* = 200%. The peak power and the energy reached a maximum of 48.5 W and 4.1 J at *v_sf_* = 200% with load = 2 kg.

For the elbow flexion, all the dynamic parameters increased with the load applied. Moreover, upon increasing the velocity of movement, the dynamic variables increased and the differences between conditions were accentuated at higher velocities. Maximum torque was lower than 11 N·m at *v_sf_* = 20% and was 16.5 N·m (0 kg), 25.7 N·m (0.5 kg), 35.1 N·m (1 kg), and 53.7 N·m (2 kg) at *v_sf_* = 200%. Peak power started from nearly 5 W for all the loading conditions and arrived at 99.6 W (0 kg), 153.7 W (0.5 kg), 208 W (1 kg), and 316.8 W (2 kg). Lastly, the energy was lower than 7 J at *v_sf_* = 20% and was 9.3 J (0 kg), 14.5 J (0.5 kg), 19.7 J (1 kg), and 30 J (2 kg) at *v_sf_* = 200%. All the results are reported in detail in Table 1.

**Figure 6:**
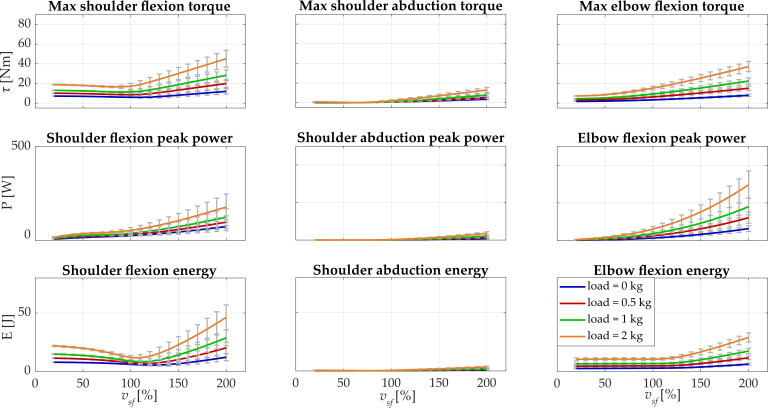
Results of the simulations when changing velocity and carried load are reported for the female subject. For each *v_sf_* and load, the means across repetitions are shown, while the standard deviations are represented with gray error bars. In the first row, the maximum torque is depicted for all the considered upper limb degrees of freedom; in the second row, the peak power is reported; the expended energy is presented in the last row. In each column, the considered degrees of freedom are represented (shoulder flexion, shoulder abduction, and elbow flexion).

In Figure 6, the recruiting curves computed for the female subject are reported.

The maximum torque had a range of 7.2–18.8 N·m at *v_sf_* = 20%, and, upon increasing the velocity, the maximum torque increased the maximum value at the highest velocity: 11.9 N·m (0 kg), 19.9 N·m (0.5 kg), 28.3 N·m (1 kg), and 45.2 N·m (2 kg). The peak power was in the range 7.2–17.9 W at *v_sf_* = 20% and reached the maximum values at *v_sf_* = 200%: 74.2 W (0 kg), 97.5 W (0.5 kg), 123.8 W (1 kg), and 178.4 W (2 kg). At *v_sf_* = 20%, the expended energy was between 7.8 J (0 kg) and 21.8 J (2 kg), and the maximum values were found at the highest velocity: 12.1 J (0 kg), 20.1 J (0.5 kg), 28.7 J (1 kg), and 46.3 J (2 kg). As for the male subject, the expended energy of the shoulder flexion had a minimum point in all the loading conditions. For load = 0 kg and load = 0.5 kg, the optimal velocity was 195.6 °/s (*v_sf_* = 120%) that corresponded to an expended energy of 5.3 J and 6.7 J; for load = 1 kg and load = 2 kg, the optimum was found at 179.3 °/s (*v_sf_* = 110%) with E = 8.1 J and E = 11.5 J. 

For the shoulder abduction, maximum torque was lower than 1 Nm for *v_sf_* = 20% in all the conditions and reached 3.5 N·m (0 kg) and 12.9 N·m (2 kg) at *v_sf_* = 200%. The peak power and the energy reached a maximum of 36.2 W and 3.4 J at *v_sf_* = 200% with load = 2 kg.

For the elbow flexion, maximum torque was lower than 8 N·m at *v_sf_* = 20% and was 7.9 N·m (0 kg), 15.1 N·m (0.5 kg), 22.5 N·m (1 kg), and 37.2 N·m (2 kg) at *v_sf_* = 200%. Peak power arrived at 63.2 W (0 kg), 121.4 (0.5 kg), 179.9 (1 kg), and 297.1 W (2 kg). Lastly, the energy was lower than 11 J at *v_sf_* = 20% and was 6.1 J (0 kg), 11.6 J (0.5 kg), 17.4 J (1 kg), and 29.1 J (2 kg) at *v_sf_* = 200%. All the results are reported in detail in Table 2.

Statistical analyses were performed on the expended energy exerted for shoulder flexion to find the velocities at which there were statistically significant differences from the optimal point (global energy minimum). For the male subject, without any carried load, the range of velocity in which the energy had no significant differences from the biomechanical optimum was the interval between *v_sf_* = 70% and *v_sf_* = 120%. For speed outside of this range, significant differences were found at *v_sf_* = 60% (*p* = 0.002) and at *v_sf_* = 130% (*p* = 0.007). With load = 0.5 kg, significant differences were found for *v_sf_* ≤ 50% (*p* = 0.004) and for *v_sf_* ≥ 130% (*p* < 0.001). For load = 1 kg, significant differences were found for *v_sf_* ≤ 50% with *p* = 0.03 and for *v_sf_* ≥ 120% with *p* = 0.001. Lastly, with load = 2 kg, significant differences were found for *v_sf_* ≤ 40 (*p* = 0.03) and for *v_sf_* ≥ 120% (*p* < 0.001). 

The female subject showed a range of velocity with no significant differences from the biomechanical optimum in the interval between *v_sf_* = 50% and *v_sf_* = 160% without any carried load. On the contrary, differences were found at *v_sf_* = 40% (*p* = 0.03) and at *v_sf_* = 160% (*p* < 0.001). With load = 0.5 kg, significant differences were found for *v_sf_* ≤ 50% (*p* = 0.046) and for *v_sf_* ≥ 160% (*p* < 0.001). For load = 1 kg, the range of velocity with no significant differences was between *v_sf_* = 50% and *v_sf_* = 140% (*p* = 0.02 at *v_sf_* = 40% and *p* = 0.02 at *v_sf_* = 150%). Lastly, with load = 2 kg, significant differences were found for *v_sf_* ≤ 40 (*p* = 0.03) and for *v_sf_* ≥ 150% (*p* < 0.001). 

**Figure 7:**
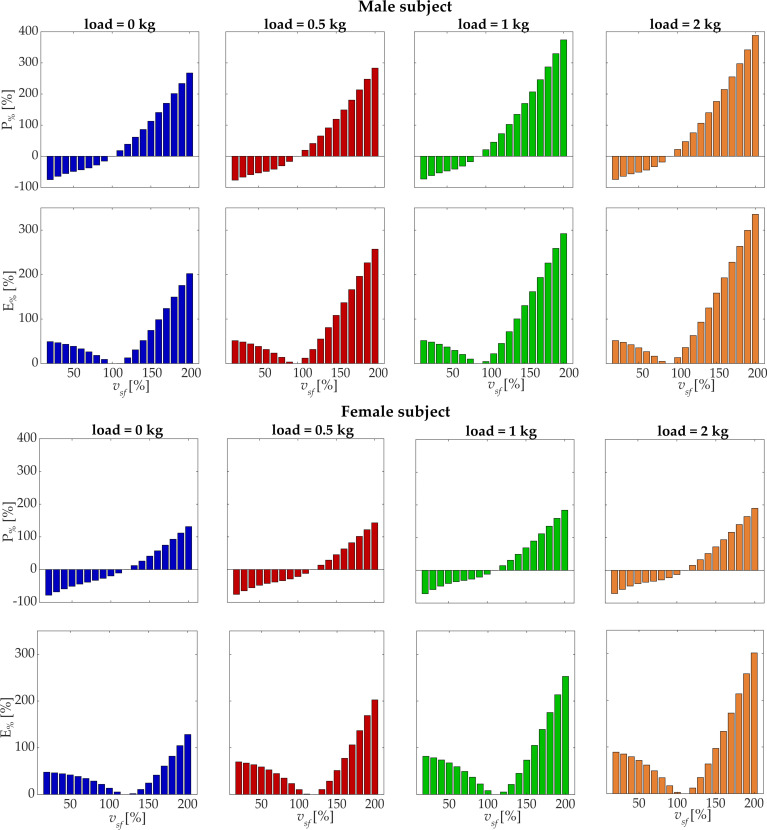
The percentage deviation of shoulder flexion *P*_%_ (power deviation) and energy *E*_%_ (energy deviation) with respect to the optimal point is reported for each loading condition. The bars represent the deviation in % with respect to the point of minimum energy for each specific loading condition. Results are reported for both the male subject (**upper panel**) and the female subject (**lower panel**).

The percentage variations of power and energy of shoulder flexion from the minimum energy speed are reported in Figure 7 for both subjects. For the male subject, when decreasing the velocity from the optimal one, *P*_%_ was nearly −75% at *v_sf_* = 20% for all the loading conditions, while *E*_%_ increased to 50%. On the contrary, when increasing the velocity, both power and energy increased, arriving at higher percentage of deviation, with differences between loading conditions. At *v_sf_* = 200%, *P*_%_ was 267% (0 kg), 283% (0.5 kg), 374% (1 kg), and 388% (2 kg), while *E*_%_ was 202% (0 kg), 257% (0.5 kg), 292% (1 kg), and 335% (2 kg). The female subject showed *P*_%_ ranging between −71% and −78% at *v_sf_* = 20% and *E*_%_ between 48% and 89%. At *v_sf_* = 200%, *P*_%_ was 131% (0 kg), 143% (0.5 kg), 184% (1 kg), and 190% (2 kg), while *E*_%_ was 128% (0 kg), 202% (0.5 kg), 253% (1 kg), and 301% (2 kg).

*E*_%_ can be considered as an indicator of the inefficiency of the movement that describes the differential energy required with respect to the optimal condition (given a specific load); high *E*_%_ indicates that the movement is highly inefficient and requires extra biomechanical effort. 

## 4. Discussion

In this study, starting from experimental data acquired with motion capture, we simulated different velocities of movement and loading conditions when performing frontal reaching movements. The simulations were carried out with a biomechanical model, maintaining the original path of recorded data and varying the temporal motion law and the carried load at the hand. We identified ranges for biomechanical efficient motion and quantified kinematics and dynamic curves when varying velocity and loads. Atkeson et al. [27] demonstrated that the trajectory is maintained when varying speed and load during vertical arm movements, and the normalized velocity profiles are invariant, even though other studies revealed that boundary conditions may alter movement kinematics [21]. Our results showed that the main degree of freedom involved in the execution of the movement and that exhibited the highest power and energy in the tested conditions was the shoulder flexion. From the energy point of view, we found that there is a velocity corresponding to a lower estimated expended energy for performing the movement. We presented data for two subjects, a male and a female, and the results were very similar. Since the inertial forces were lower, the female showed smaller values of torques, power, and energy, and the minimum energy speed was higher with respect to the male. Therefore, the results could depend on the anthropometry of the subject, suggesting that general trends are found across subjects, but that subject-specific analysis might be needed.

Interestingly, the “minimum energy velocity”, when external loads are not carried, was very similar to the velocity profile selected by the user while performing the movement during the experimental acquisition. As shown by Caimmi et al. [28], movements at self-selected natural speed are characterized by a balance between kinetic and potential energies. For lower speeds, the kinetic energy is limited and the resulting torque required is higher, leading to an increase in the co-contractions of muscles to stabilize the movement; on the contrary, a fastest movement would be characterized by a higher kinetic energy, leading to a large torque required to decelerate the movement. It is, thus, not surprising that the recorded natural movements were performed at biomechanically optimal speed.

As expected, when increasing movement speed and, consequently, the acceleration, the dynamic effort requested is higher due to the higher inertia. The higher inertia affects both agonist and antagonist muscles as demonstrated by the presence of negative components of torque and power, necessary for controlling the deceleration of the movement and stabilizing the joints [29]. When scaling down the speed of the movement below the optimal velocity, the expended energy increases even if the inertia is lower because of the presence of gravitational forces that act for a longer time. Thus, both gravity and inertial forces influence the movement in relation to the speed [30]. Moreover, our results showed that the optimal speed decreases when a load is carried by the subject. This is due to the higher inertia at the hand; therefore, the equilibrium between gravitational and inertial forces is at a lower velocity [31]. 

Due to the increasing interest in the physical and psychological state of workers, these results can be useful for improving the human–robot collaboration in industrial contexts. Other studies found that less physical and mental effort is required when the assistive robot moves with biological velocity patterns [32,33]. Work fatigue is composed of three different levels of fatigue: emotional, mental, and physical fatigue and may affect psychological and physical health of the workers leading to less efficient work recovery and negative work attitude. [34]. Therefore, an adequate physical demand is fundamental to preserve the mental state of the worker. It has also been demonstrated with electroencephalographic analysis that physical fatigue can enhance the mental fatigue as a function of brain activation, functional connection, and complexity [35]. According to our results, the collaborative robot can be set in order to maintain the range of optimal speed for the worker, preventing physical fatigue and preserving the mental stress, depending on the carried load. 

Furthermore, a biomechanical inefficiency index (*E*_%_) is suggested, able to quantify the amount of additional energy required if the movement is not performed in the optimal biomechanical condition; a higher index denotes a more inefficient movement (requiring higher energy). Our findings can also be the basis for a structured index of fatigue based on biomechanical quantities, similar to fatigue indices based on joint capacity [36] and on torque generated at the joint level [37,38]. Other studies quantified the level of fatigue as the ratio between the initial and final value of biomechanical quantities such as torque [39], angular position [40], and time to peak velocity [41] over a repetitive task. The applicability of this method has been demonstrated only on a cyclic movement, but the same approach can be extended and the indices can be scaled to other domains of applications where continuous movements are not segmented in cyclic phases, such as in the industry scenario. Another possible application could be in the rehabilitation field; during robotic training, the application of comfortable speed requiring less effort can help the patient to feel less stressed and to improve motor recovery [42]. Further applications might consider more variable conditions.

A limitation of this work is the assumption that the kinematic profiles do not change when varying load and speed. This assumption is reasonable considering the employed standardized movement, but other studies showed that, when modifying the conditions in which the movement is performed, the kinematics changes so that the energic expenditure is minimized. Other authors found that the kinematics is modulated by torques [23], and that dynamics override kinematics in vertical movements [43]. Our results are not in contrast with these observations since we found that kinematics adapts to dynamics (the optimal speed decreased with a higher carried load). However, the validity of our analysis is limited to reaching movements performed with the same velocity profiles, and our results cannot be generalized to every scenario. In future works, the kinematic changes due to effect of load and speed will be considered.

Another limitation of this work is to implicitly consider only cyclic movements. In addition, the effect of the fatigue on the movement was not considered. Future developments could be the extension of this method to other upper-limb movements and the validation of the results with experimental tests on a cohort of subjects and during real interaction in a working scenario. Moreover, it could be interesting to correlate these results with electromyographic measures [29] in order to evaluate how the motor control is affected by movement speed and carried load and how the central nervous system manages these variables [44,45]. Lastly, more movement speeds could be added in order to tighten the curves and to better define the optimal range of velocity. 

## 5. Conclusions

In this study, we analyzed the influence of the movement velocity and carried load on the performance of the upper limb. To do this, we performed simulations with a biomechanical model starting from experimental data. Our results suggested that both the velocity and the load influenced the effort required to execute the movement; moreover, there exists a range of velocity in which the expended energy at shoulder flexion degree of freedom is minimum. Lastly, we introduced a preliminary inefficiency index to quantify the energy deviation from the optimal point. Future developments could be the inclusion of more gestures and the integration of electromyographic measures.

## Figures and Tables

**Table 1:** Values of torque (*τ*), power (*P*), and energy (*E*) are reported for each *v_sf_* in all the loading conditions for each degree of freedom for the male subject.

		*v_sf_* (%)																		
	Load (kg)	20	30	40	50	60	70	80	90	100	110	120	130	140	150	160	170	180	190	200
Shoulder Flexion																				
*τ* (N·m)	0	13.1	12.9	12.8	12.6	12.3	11.9	11.6	11.7	13.0	14.4	16.1	17.9	19.7	21.5	23.3	25.1	26.9	28.7	30.5
	0.5	16.1	15.9	15.74	15.5	15.1	14.6	14.4	15.7	17.8	20.1	22.5	25.0	27.6	30.2	32.8	35.3	37.9	40.5	43.0
	1	19.1	19.0	18.7	18.4	17.9	17.5	17.7	20.0	22.7	25.8	28.9	32.2	35.5	38.9	42.3	45.6	49.0	52.3	55.6
	2	25.2	25.0	24.6	24.2	23.5	23.1	24.9	28.5	32.7	37.2	41.8	46.6	51.5	56.4	61.3	66.3	71.2	76.1	80.9
*P* (W)	0	11.5	16.5	20.5	23.6	26.1	28.9	33.2	39.1	46.3	54.7	64.2	74.7	86.1	98.4	111.4	125.1	139.5	154.6	170.2
	0.5	14.1	20.0	24.7	28.1	31.1	35.4	41.9	50.4	60.6	72.3	85.5	100.1	115.9	132.8	150.9	170.0	189.9	210.7	232.3
	1	16.7	23.5	28.9	32.7	36.5	42.5	51.1	62.1	75.3	90.3	107.2	125.8	145.9	167.6	190.7	215.1	240.5	267.0	294.6
	2	21.9	30.7	37.3	42.1	47.9	57.1	70.0	86.0	104.9	126.6	150.8	177.4	206.4	237.6	270.6	305.4	341.9	380.2	420.0
*E* (J)	0	12.7	12.5	12.2	11.8	11.3	10.7	10.1	9.3	8.5	8.6	9.6	11.1	12.9	14.9	16.9	19.1	21.2	23.5	25.7
	0.5	15.8	15.5	15.1	14.5	13.7	12.9	11.9	10.8	10.4	11.7	13.8	16.2	18.9	21.8	24.7	27.8	30.9	34.1	37.3
	1	18.9	18.5	17.9	17.1	16.2	15.0	13.7	12.5	13.0	15.2	18.1	21.4	25.0	28.8	32.7	36.6	40.7	44.8	48.9
	2	25.1	24.5	23.6	22.4	21.0	19.3	17.4	16.6	18.8	22.5	27.0	32.0	37.3	42.9	48.6	54.4	60.3	66.3	72.3
**Shoulder Abduction**																				
*τ* (N·m)	0	4.8	4.8	4.8	5.0	5.3	5.8	6.3	6.9	7.5	8.1	8.7	9.4	10.0	10.6	11.2	11.8	12.4	13.0	13.6
	0.5	5.6	5.5	5.6	6.0	6.5	7.1	7.8	8.5	9.3	10.1	10.9	11.7	12.6	13.4	14.2	15.0	15.8	16.6	17.3
	1	6.3	6.3	6.5	7.0	7.6	8.3	9.2	10.1	11.0	12.0	13.0	14.1	15.1	16.2	17.2	18.2	19.2	20.2	21.1
	2	7.8	7.9	8.3	8.9	9.8	10.8	12.0	13.2	14.6	15.9	17.4	18.8	20.3	21.7	23.2	24.6	26.0	27.4	28.7
*P* (W)	0	1.1	1.7	2.3	2.9	3.6	4.3	5.2	6.1	7.1	8.2	9.4	10.7	12.0	13.5	15.0	16.6	18.2	19.9	21.7
	0.5	1.3	2.0	2.7	3.5	4.3	5.3	6.3	7.5	8.9	10.3	11.9	13.6	15.4	17.3	19.3	21.5	23.7	26.0	28.3
	1	1.5	2.3	3.1	4.0	5.1	6.2	7.5	9.0	10.6	12.4	14.4	16.5	18.8	21.2	23.7	26.3	29.1	32.0	35.0
	2	1.9	2.9	4.0	5.2	6.6	8.1	9.9	11.9	14.2	16.6	19.3	22.3	25.5	28.8	32.4	36.2	40.1	44.2	48.5
*E* (J)	0	1.1	1.1	1.1	1.2	1.2	1.2	1.3	1.3	1.4	1.4	1.4	1.5	1.5	1.6	1.6	1.7	1.8	1.8	1.9
	0.5	1.3	1.3	1.4	1.4	1.4	1.5	1.5	1.6	1.7	1.7	1.8	1.9	1.9	2.0	2.1	2.2	2.2	2.3	2.4
	1	1.5	1.6	1.6	1.6	1.7	1.7	1.8	1.9	2.0	2.1	2.1	2.2	2.3	2.4	2.5	2.6	2.7	2.8	3.0
	2	2.0	2.0	2.1	2.1	2.2	2.3	2.4	2.5	2.6	2.7	2.8	3.0	3.1	3.2	3.4	3.6	3.7	4.0	4.1
**Elbow Flexion**																				
*τ* (N·m)	0	3.7	3.9	4.1	4.4	4.8	5.4	6.0	6.8	7.5	8.3	9.1	10.0	10.9	11.8	12.7	13.7	14.6	15.5	16.5
	0.5	5.4	5.6	6.0	6.5	7.2	8.1	9.1	10.2	11.5	12.8	14.1	15.5	16.9	18.4	19.8	21.3	22.8	24.3	25.7
	1	7.1	7.4	7.9	8.6	9.6	10.8	12.2	13.8	15.5	17.2	19.1	21.0	23.0	25.0	27.0	29.0	31.0	33.0	35.1
	2	10.6	11.0	11.7	12.8	14.4	16.3	18.5	20.9	23.5	26.3	29.1	32.1	35.1	38.1	41.2	44.3	47.5	50.6	53.7
*P* (W)	0	2.1	3.4	4.9	6.7	8.9	11.6	14.8	18.6	22.9	27.9	33.4	39.6	46.4	53.8	61.8	70.4	79.5	89.3	99.6
	0.5	3.1	5.0	7.2	9.9	13.3	17.4	22.3	28.1	34.9	42.5	51.2	60.7	71.3	82.8	95.2	108.5	122.7	137.8	153.7
	1	4.1	6.6	9.5	13.2	17.7	23.2	29.9	37.7	46.8	57.2	68.9	82.0	96.3	111.8	128.6	146.7	165.9	186.4	208.0
	2	6.1	9.8	14.2	19.7	26.5	34.9	45.0	57.0	70.8	86.7	104.6	124.4	146.2	169.9	195.6	223.1	252.5	283.9	316.8
*E* (J)	0	2.4	2.5	2.6	2.8	3.0	3.2	3.4	3.6	3.9	4.3	4.8	5.3	5.8	6.4	6.9	7.54	8.1	8.7	9.3
	0.5	3.5	3.7	3.9	4.1	4.4	4.7	5.1	5.4	5.9	6.5	7.3	8.1	9.0	9.9	10.8	11.7	12.6	13.6	14.5
	1	4.7	4.9	5.1	5.4	5.8	6.2	6.7	7.2	7.9	8.8	9.9	11.0	12.2	13.4	14.6	15.9	17.1	18.4	19.7
	2	7.0	7.2	7.6	8.1	8.7	9.3	10.1	10.8	11.9	13.4	15.0	16.7	18.5	20.4	22.3	24.2	26.1	28.1	30.0

**Table 2:** Values of torque (*τ*), power (*P*), and energy (*E*) are reported for each *v_sf_* in all the loading conditions for each degree of freedom for the female subject.

		*v_sf_* (%)																		
	load (kg)	20	30	40	50	60	70	80	90	100	110	120	130	140	150	160	170	180	190	200
Shoulder Flexion																				
*τ* (N·m)	0	7.2	7.1	7.0	6.9	6.7	6.5	6.3	6.1	6.0	5.9	6.1	6.6	7.3	8.1	8.8	9.6	10.4	11.2	11.9
	0.5	10.1	10.0	9.8	9.7	9.4	9.1	8.8	8.6	8.6	8.8	9.6	10.7	12.0	13.3	14.6	15.9	17.3	18.6	19.9
	1	13.0	12.9	12.7	12.4	12.1	11.8	11.4	11.2	11.3	12.0	13.4	15.2	17.0	18.8	20.7	22.6	24.5	26.4	28.3
	2	18.8	18.6	18.3	18.0	17.5	17.0	16.6	16.6	17.1	18.9	21.5	24.3	27.2	30.1	33.1	36.1	39.2	42.2	45.2
*P* (W)	0	7.0	10.2	13.1	15.7	17.8	19.7	21.5	23.4	25.8	28.7	32.1	36.0	40.4	45.3	50.5	56.0	61.8	67.9	74.2
	0.5	9.70	14.0	17.8	20.9	23.1	24.8	26.5	28.6	31.5	35.4	40.1	45.6	51.7	58.4	65.5	73.1	80.8	89.0	97.5
	1	12.4	17.9	22.6	26.1	28.5	30.1	31.9	34.6	38.5	43.6	49.9	57.1	65.0	73.6	82.7	92.4	102.5	113.0	123.8
	2	17.9	25.7	32.0	36.5	39.3	41.1	43.5	47.6	53.6	61.6	71.0	81.6	93.2	105.8	119.2	133.2	147.8	162.8	178.4
*E* (J)	0	7.8	7.7	7.6	7.5	7.3	7.1	6.8	6.5	6.0	5.6	5.3	5.4	5.9	6.6	7.5	8.5	9.6	10.8	12.1
	0.5	11.3	11.1	10.9	10.6	10.2	9.6	9	8.2	7.3	6.7	6.7	7.4	8.5	10.1	11.8	13.7	15.7	17.9	20.1
	1	14.8	14.5	14.1	13.6	13.0	12.1	11.1	10.0	8.8	8.1	8.6	9.9	11.8	14.1	16.7	19.5	22.4	25.5	28.7
	2	21.8	21.3	20.7	19.8	18.6	17.2	15.5	13.5	11.9	11.5	12.9	15.5	18.9	22.7	26.9	31.4	36.2	41.1	46.3
**Shoulder Abduction**																				
*τ* (N·m)	0	0.1	0.1	0.05	0.03	0.1	0.2	0.3	0.5	0.7	0.9	1.2	1.4	1.7	2.0	2.3	2.6	2.9	3.2	3.5
	0.5	0.3	0.2	0.2	0.1	0.1	0.1	0.2	0.5	0.8	1.1	1.5	1.9	2.4	2.8	3.3	3.8	4.3	4.9	5.4
	1	0.5	0.4	0.3	0.2	0.1	0.1	0.3	0.6	1.1	1.6	2.1	2.7	3.4	4.1	4.8	5.5	6.2	7.0	7.7
	2	0.9	0.8	0.6	0.4	0.2	0.2	0.6	1.3	2.0	2.9	3.8	4.9	5.9	7.0	8.2	9.4	10.5	11.7	12.9
*P* (W)	0	0.01	0.02	0.03	0.1	0.1	0.1	0.2	0.4	0.6	0.9	1.3	1.8	2.4	3.1	3.8	4.6	5.6	6.6	7.6
	0.5	0.01	0.02	0.04	0.1	0.1	0.2	0.3	0.5	0.9	1.5	2.2	3.1	4.2	5.4	6.8	8.40	10.1	12.0	13.9
	1	0.02	0.03	0.1	0.1	0.1	0.2	0.4	0.7	1.4	2.3	3.5	4.9	6.5	8.4	10.5	12.8	15.4	18.1	21.1
	2	0.03	0.05	0.1	0.1	0.2	0.4	0.7	1.5	2.7	4.4	6.4	8.8	11.6	14.7	18.3	22.2	26.5	31.2	36.2
*E* (J)	0	0.2	0.2	0.2	0.2	0.2	0.2	0.2	0.2	0.2	0.2	0.3	0.3	0.4	0.4	0.5	0.6	0.6	0.7	0.8
	0.5	0.3	0.3	0.2	0.2	0.2	0.2	0.2	0.2	0.2	0.3	0.4	0.5	0.6	0.7	0.8	0.9	1.1	1.2	1.3
	1	0.4	0.4	0.3	0.3	0.2	0.2	0.2	0.2	0.3	0.4	0.6	0.7	0.9	1.0	1.2	1.4	1.6	1.8	2.0
	2	0.6	0.5	0.5	0.4	0.3	0.2	0.2	0.3	0.5	0.7	1.0	1.2	1.5	1.8	2.1	2.4	2.7	3.1	3.4
**Elbow Flexion**																				
*τ* (N·m)	0	1.8	1.8	1.9	2.0	2.2	2.4	2.7	3.0	3.3	3.7	4.1	4.5	5.0	5.4	5.9	6.4	6.9	7.4	7.9
	0.5	3.1	3.2	3.4	3.7	4.0	4.5	5.0	5.6	6.3	7.0	7.80	8.60	9.50	10.3	11.3	12.2	13.2	14.1	15.1
	1	4.5	4.7	4.9	5.3	5.8	6.5	7.3	8.3	9.3	10.4	11.5	12.8	14.0	15.4	16.7	18.1	19.6	21.0	22.5
	2	7.3	7.5	7.9	8.6	9.5	10.7	12.1	13.6	15.3	17.1	19.1	21.1	23.2	25.5	27.7	30.0	32.4	34.8	37.2
*P* (W)	0	1.1	1.8	2.6	3.6	4.9	6.4	8.2	10.4	12.9	15.9	19.3	23.1	27.4	32.2	37.4	43.2	49.4	56.0	63.2
	0.5	2.1	3.4	4.9	6.8	9.1	12.0	15.5	19.8	24.7	30.4	37.0	44.4	52.7	61.9	72.0	83.0	94.9	107.7	121.4
	1	3.0	4.9	7.1	9.9	13.4	17.7	22.9	29.2	36.6	45.1	54.9	65.9	78.3	91.9	106.8	123.1	140.7	159.6	179.9
	2	4.9	7.9	11.6	16.2	21.9	29.1	37.7	48.1	60.3	74.5	90.7	109.0	129.4	151.9	176.6	203.5	232.5	263.7	297.1
*E* (J)	0	2.5	2.5	2.5	2.5	2.6	2.6	2.7	2.7	2.7	2.8	2.9	3.1	3.3	3.7	4.1	4.6	5.1	5.6	6.1
	0.5	4.4	4.5	4.5	4.5	4.6	4.6	4.6	4.6	4.7	4.7	4.9	5.3	5.9	6.7	7.6	8.5	9.5	10.5	11.6
	1	6.4	6.4	6.5	6.5	6.6	6.6	6.6	6.6	6.6	6.7	7.0	7.7	8.7	9.9	11.3	12.7	14.2	15.8	17.4
	2	10.3	10.4	10.4	10.5	10.5	10.6	10.6	10.5	10.5	10.7	11.4	12.7	14.5	16.6	18.9	21.3	23.8	26.4	29.1

## Data Availability

The data presented in this study are available on reasonable request from the corresponding author.
